# Emergency Department and Hospital Utilization Among Older Adults Before and After Identification of Elder Mistreatment

**DOI:** 10.1001/jamanetworkopen.2022.55853

**Published:** 2023-02-14

**Authors:** Tony Rosen, Hao Zhang, Katherine Wen, Sunday Clark, Alyssa Elman, Philip Jeng, Daniel Baek, Yiye Zhang, Zach Gassoumis, Nicole Fettig, Karl Pillemer, Mark S. Lachs, Yuhua Bao

**Affiliations:** 1 Department of Emergency Medicine, Weill Cornell Medical College/NewYork-Presbyterian Hospital, New York; 2 Department of Population Health Sciences, Weill Cornell Medicine, New York, New York; 3 Center for Gerontology and Healthcare Research, Brown University School of Public Health, Providence, Rhode Island; 4 Department of Family Medicine, University of Southern California Keck School of Medicine, Los Angeles; 5 WRMA Inc, Rockville, Maryland; 6 College of Human Ecology, Cornell University, Ithaca, New York; 7 Division of Geriatrics and Palliative Medicine, Weill Cornell Medical College/NewYork-Presbyterian Hospital, New York

## Abstract

**Importance:**

Elder mistreatment is common and has serious health consequences. Little is known, however, about patterns of health care utilization among older adults experiencing elder mistreatment.

**Objective:**

To examine emergency department (ED) and hospital utilization of older adults experiencing elder mistreatment in the period surrounding initial mistreatment identification compared with other older adults.

**Design, Setting, and Participants:**

This retrospective case-control study used Medicare insurance claims to examine older adults experiencing elder mistreatment initially identified between January 1, 2003, and December 31, 2012, and control participants matched on age, sex, race and ethnicity, and zip code. Statistical analysis was performed in April 2022.

**Main Outcomes and Measures:**

We used multiple measures of ED and hospital utilization patterns (eg, new and return visits, frequency, urgency, and hospitalizations) in the 12 months before and after mistreatment identification. Data were adjusted using US Centers for Medicare and Medicaid Services Hierarchical Condition Categories risk scores. Chi-squared tests and conditional logistic regression models were used for data analyses.

**Results:**

This study included 114 case patients and 410 control participants. Their median age was 72 years (IQR, 68-78 years), and 340 (64.9%) were women. Race and ethnicity were reported as racial or ethnic minority (114 [21.8%]), White (408 [77.9%]), or unknown (2 [0.4%]). During the 24 months surrounding identification of elder mistreatment, older adults experiencing mistreatment were more likely to have had an ED visit (77 [67.5%] vs 179 [43.7%]; adjusted odds ratio [AOR], 2.95 [95% CI, 1.78-4.91]; *P* < .001) and a hospitalization (44 [38.6%] vs 108 [26.3%]; AOR, 1.90 [95% CI, 1.13-3.21]; *P* = .02) compared with other older adults. In addition, multiple ED visits, at least 1 ED visit for injury, visits to multiple EDs, high-frequency ED use, return ED visits within 7 days, ED visits for low-urgency issues, multiple hospitalizations, at least 1 hospitalization for injury, hospitalization at multiple hospitals, and hospitalization for ambulatory care sensitive conditions were substantially more likely for individuals experiencing elder mistreatment. The rate of ED and hospital utilization for older adults experiencing elder mistreatment was much higher in the 12 months after identification than before, leading to more pronounced differences between case patients and control participants in postidentification utilization. During the 12 months after identification of elder mistreatment, older adults experiencing mistreatment were particularly more likely to have had high-frequency ED use (12 [10.5%] vs 8 [2.0%]; AOR, 8.23 [95% CI, 2.56-26.49]; *P* < .001) and to have visited the ED for low-urgency issues (12 [10.5%] vs 8 [2.0%]; AOR, 7.33 [95% CI, 2.54-21.18]; *P* < .001).

**Conclusions and Relevance:**

In this case-control study of health care utilization, older adults experiencing mistreatment used EDs and hospitals more frequently and with different patterns during the period surrounding mistreatment identification than other older adults. Additional research is needed to better characterize these patterns, which may be helpful in informing early identification, intervention, and prevention of elder mistreatment.

## Key Points

**Question:**

Do older adults experiencing elder mistreatment use the emergency department (ED) and hospital more or differently in the period surrounding initial mistreatment identification compared with other older adults?

**Findings:**

In this case-control study examining Medicare claims for 114 older adults known to be experiencing elder mistreatment compared with 410 matched control participants, those experiencing elder mistreatment had nearly 3 times the odds of receiving care in the ED and nearly twice the odds of being hospitalized.

**Meaning:**

These findings suggest that older adults experiencing elder mistreatment use EDs and hospitals more frequently and with different patterns during the period surrounding mistreatment identification compared with other older adults.

## Teaser

This case-control study uses Medicare claims data to examine emergency department and hospital utilization among older adults experiencing elder mistreatment during the period surrounding initial mistreatment identification compared with that of other older adults.

## Introduction

Elder mistreatment, including physical abuse, sexual abuse, neglect, psychological abuse, or financial exploitation, is common and has serious medical and social consequences.^1^ Elder mistreatment can have devastating consequences for older adults and families, increasing isolation, depression, and even mortality.^1^ This mistreatment is dramatically underrecognized and underreported, however.^1^ For many older adults, assessment by health care practitioners may represent the only contact outside their home. Therefore, clinicians have a unique opportunity to identify suspected mistreatment and initiate intervention.^2,3,4,5,6,7,8^ Influential research in child abuse^9,10,11,12,13,14^ and intimate partner violence^15,16^ has focused on health care utilization before identification, highlighting that many survivors had multiple previous visits for likely abuse-related issues, suggesting “missed opportunities” for identification and early intervention. Previous research has shown that older adults experiencing mistreatment have increased rates of emergency department (ED) use^2,5^ and hospitalization^17^ but have not further characterized this utilization or examined its association with mistreatment detection.

We have proposed a conceptual model to explain this increased utilization and have described it in detail elsewhere.^18^ Briefly, we hypothesize that because of isolation, poor connection to outpatient and/or primary care, and medical issues directly related to mistreatment, older adults experiencing mistreatment are more likely to use the ED and be hospitalized before and after mistreatment identification. In addition, they are more likely to present to the ED for injuries as well as nonurgent issues and ambulatory care sensitive conditions (ACSCs)^19^ for which hospitalization could be prevented by primary care interventions.

Furthermore, older adults experiencing mistreatment are more likely to seek treatment at multiple EDs and hospitals and to have readmissions to EDs or hospitals within short intervals. Whether this conceptual model consistently describes health care utilization by older adults experiencing mistreatment has not been assessed, however, and little is known about the longitudinal features of ED and hospital utilization in this population, particularly surrounding initial identification of mistreatment.^20,21^

In this study, we assessed ED and hospital utilization of older adults known to be experiencing elder mistreatment in the time period surrounding their initial mistreatment identification and compared these patterns with health care utilization of other older adults.

## Methods

This retrospective case-control study was approved by the Weill Cornell Institutional Review Board and informed consent was waived per the Common Rule. This work is reported based on the Strengthening the Reporting of Observational Studies in Epidemiology (STROBE) reporting guideline.

### Study Design and Participants

We used Medicare insurance claims to examine ED and hospital utilization patterns among older adults experiencing mistreatment in the 12 months before and 12 months after initial mistreatment identification compared with matched control participants who had no documented exposure. Details of our approach have been published elsewhere.^18^ Briefly, we examined cohorts of older adults experiencing mistreatment who were from Brooklyn, New York,^22^ and Seattle, Washington.^23^ These cohorts included a total of 503 case patients initially identified between January 1, 2003, and December 31, 2012. The cases, which have been used in research previously,^22,23^ are unique: as the perpetrators have pled guilty or been convicted, the occurrence of elder mistreatment has been verified. Also, for each case, the date when the mistreatment initially was identified by the authorities is known. Most commonly, initial identification occurred when the older adult experiencing mistreatment or another person called 911. The Brooklyn cohort includes only cases of physical abuse, while the Seattle cohort includes cases of physical abuse and other types of mistreatment.

### Linkage of Case Patients to Medicare Claims Data

**Figure 1.**
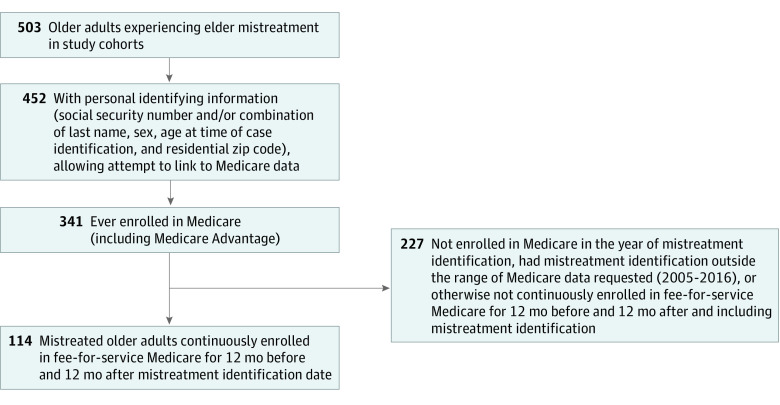
Linkage of Known Elder Mistreatment Cases From Study Cohorts From Brooklyn, New York, and Seattle, Washington, to Medicare Fee-for-Service Data

We attempted to link the case patients to fee-for-service Medicare claims to examine health care utilization. Fee-for-service Medicare is the largest repository of health care data for older adults in the US,^24^ offering comprehensive information about utilization of health care services. To link the case patients to claims, we used social security number and/or a combination of sex, last name, date of birth, and residential zip code. A higher percentage of case patients for which we were able to provide the social security number successfully linked. Of all cases that did not link to Medicare data, 42 patients were younger than 65 years 1 year after mistreatment identification. Among older adults successfully linked, we identified those continuously enrolled in Medicare fee-for-service Parts A and B for 12 calendar months before the month of initial mistreatment identification and 12 months after and including the month of initial mistreatment identification. The resulting sample contained 114 case patients (22.7%). Details of linking are shown in Figure 1.

### Identification of ED Visits and Hospitalizations in Medicare Claims

To identify ED visit events, we used both outpatient and inpatient Medicare claims, which include codes to facilitate billing. Any claim was considered an ED visit if it had a *Current Procedural Technology* (*CPT*) code of 99281 to 99285 or a Revenue Center Code of 0450 to 0459 or 0981. Any ED visit found on outpatient claims was considered a treat-and-release ED visit, while a visit found on inpatient claims was considered an ED visit that led to hospital admission.

To identify hospitalizations, we used the Medicare Provider Analysis and Review file, which includes information on all inpatient stays. We excluded skilled nursing facility, long-term acute care hospital, and inpatient rehabilitation facility stays.

### Measures to Assess ED and Hospital Utilization

We used several measures to assess ED and hospital utilization.^18^ For ED utilization, these included at least 1 ED visit, multiple ED visits, at least 1 ED visit for injury, visits to multiple EDs, high-frequency ED use (≥4 visits over 12 months^25,26,27,28^), visits for ACSCs, and low-urgency visits (identified using Medicare *CPT* billing codes 99281 and 99282, indicating low severity and no additional procedures billed^29,30^).

We also examined the occurrence of return ED visits, identifying all “new” ED visits (ie, those without a preceding visit in the previous 30 days) and assessing the presence of another ED visit within 3 or 7 days of the index visit. We did not consider index visits occurring in the last 7 days of the study period for each patient. We also examined the percentage of ED visits that led to hospitalization.

To measure hospital utilization, we used at least 1 hospitalization, multiple hospitalizations, at least 1 hospitalization for injury, hospitalization at multiple hospitals, and hospitalization with an ACSC.

Emergency department visits or hospitalizations for injury were identified using the primary diagnoses assigned for the health care encounter, with codes 800 to 999 in the *International Classification of Diseases, Ninth Revision* (*ICD-9*) and codes from Chapter 19 beginning with S or T in the *International Classification of Diseases, Tenth Revision* (*ICD-10*),^31,32^ similar to previous work in child abuse,^33,34^ but excluding codes that did not actually represent injuries based on research team review.

Visits to multiple EDs and hospitalizations at multiple facilities were identified using facility codes to identify each ED or hospital providing services to an individual older adult. To ensure that we did not incorrectly consider interhospital transfers as discrete hospitalizations at different hospitals, we excluded hospitalizations at a different facility that started on the same day or the next day after a hospital discharge when measuring multiple hospitalizations or hospitalization at multiple hospitals.

Emergency department visits or hospitalizations for ACSCs were identified by primary diagnoses for 1 of the 11 ACSCs established for Medicare data: cellulitis, asthma, chronic obstructive pulmonary disease, congestive heart failure, dehydration, pneumonia, septicemia, stroke, urinary tract infection, acute diabetic event, and lower limb peripheral vascular disease among patients with diabetes.^35^

### Period Examined Relative to Mistreatment Identification

To provide additional insight about the pattern of utilization and its association with mistreatment identification date, we subdivided the 24-month period for each case into 6 equal-length subperiods of 4 months. These periods represented chronic preidentification, subacute preidentification, acute preidentification, identification/acute postidentification, subacute postidentification, and chronic postidentification. Differences in utilization during these periods may improve understanding and offer opportunities for earlier identification of elder mistreatment. For each of these subperiods, we examined ED visits per patient, the percentage of patients with at least 1 ED visit, hospital inpatient days per patient, and patients with at least 1 hospitalization.

### Control Participants

We selected from fee-for-service Medicare data a maximum of 4 control participants per case patient, matched on age (±2 years), sex, race and ethnicity, and residential zip code. Race and ethnicity were reported based on the race variable in the Medicare enrollment file and recoded into 3 categories (racial or ethnic minority, White, or unknown). We matched on these demographic criteria because of their known correlation with variations in health care utilization. Each control participant for a case patient was required to have had both 12 months of continuous enrollment before and 12 months after and including the month containing the case patient’s mistreatment identification date.

### Adjustment for Hierarchical Condition Category Score

To ensure comparability between case patients and control participants on underlying health conditions as well as other risks of health care utilization, we adjusted comparisons between groups using US Centers for Medicare and Medicaid Services Hierarchical Condition Categories (CMS-HCC) risk scores.^36,37,38,39^ The CMS-HCC risk scores were designed to predict future health care costs for Medicare beneficiaries and use combinations of *ICD* diagnostic codes in Medicare claims and demographic variables, including age, sex, Medicaid eligibility, and disability.^36^ Multiple risk scores may be calculated (community, institutional, new enrollees, and Special Needs Plan new enrollees) and the algorithms used are updated each year.

We decided to adjust for, rather than match on, HCC score because we were concerned about potential overmatching. Also, we recognized that with a relatively small sample, increasing the number of factors on which we matched would impair our ability to analyze case patients because of inability to find a control participant.

For this study, we used the community risk score. For each case patient and matched control participant, we calculated the CMS-HCC risk score using data from the 12 months before mistreatment identification. For case patients with a mistreatment identification date between July 1 and December 31, we used the CMS algorithm pertaining to the calendar year of the mistreatment identification date; for cases with a mistreatment identification date between January 1 and June 30, we used the algorithm pertaining to the calendar year prior to mistreatment identification.

### Statistical Analysis

We used 2-sided χ^2^ tests to compare differences in outcomes between the elder mistreatment group and the control group; *P* < .05 was considered statistically significant. For comparisons with a sample size in at least 1 cell of an expected value less than 5, Fisher exact tests were used instead. We used conditional logistic regression to take into account matching and generate adjusted odds ratios (AORs). We present data as frequencies with proportions and AORs with 95% CIs.

Analysis was conducted within the CMS Virtual Research Data Center, using SAS Enterprise, version 7.1 (SAS Institute). Statistical analysis was performed in April 2022.

## Results

**Figure 2.**
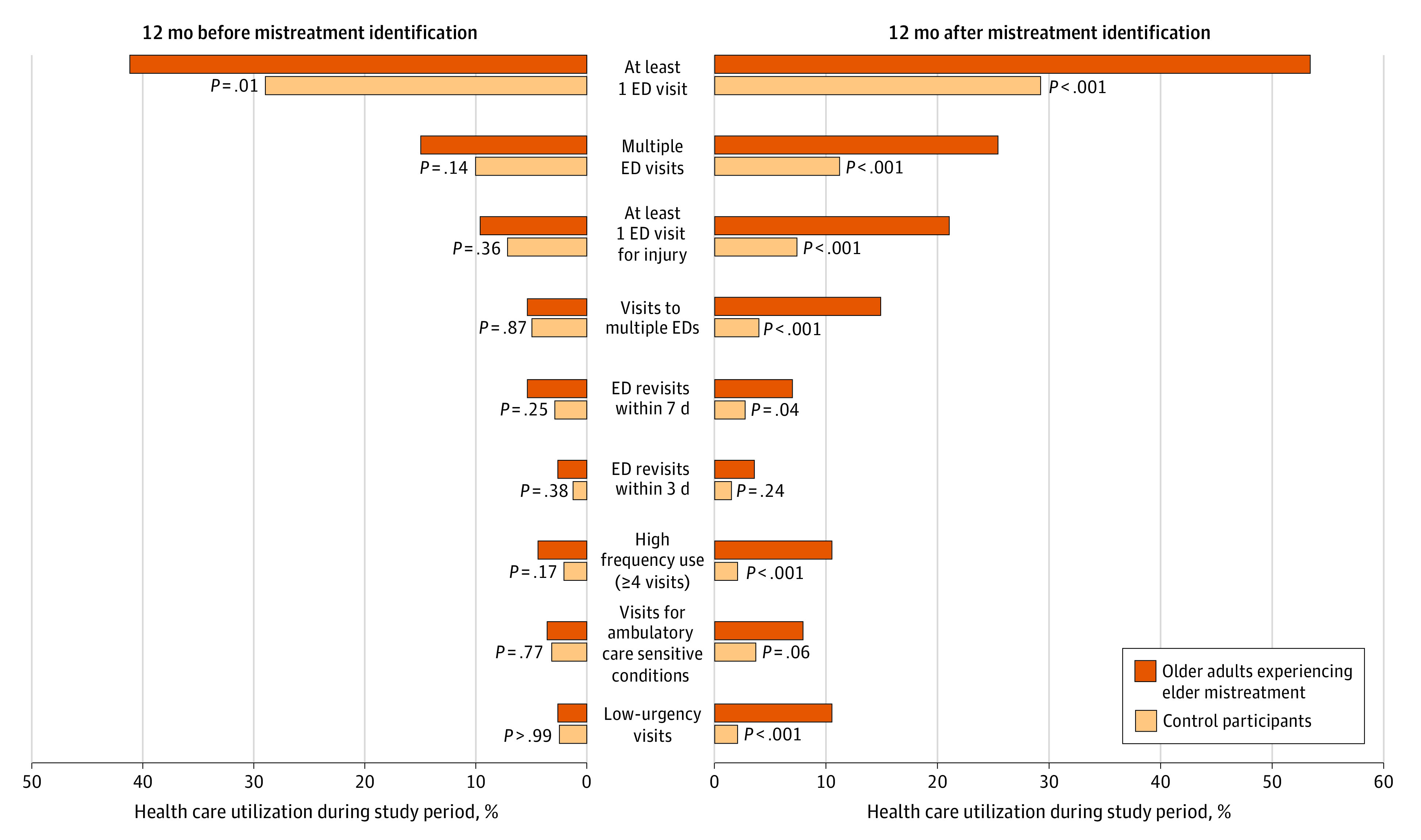
Emergency Department Utilization Among 114 Older Adults Known to Be Experiencing Elder Mistreatment Compared With 410 Matched Control Participants ED indicates emergency department.

We examined in detail ED and hospital utilization for 114 case patients who had experienced elder mistreatment and 410 matched control participants over the 24 months surrounding mistreatment identification. Their median age was 72 years (IQR, 68-78 years); 340 (64.9%) were women and 184 (35.1%) were men. Race and ethnicity were reported as racial or ethnic minority (114 [21.8%]), White (408 [77.9%]), or unknown (2 [0.4%]). Among the 114 case patients, 104 (91.2%) were community dwelling, 1 (0.9%) lived in an adult family home, and 5 (4.4%) lived in senior living, retirement centers, or assisted living; residence type was unknown for 4 individuals (3.5%). Figure 2 visually depicts a comparison of 9 measures of ED utilization in the 12-month periods before and after initial mistreatment identification. It shows that in periods before and after mistreatment identification, older adults experiencing mistreatment were significantly more likely to have visited the ED. For all measures of ED utilization, older adults experiencing mistreatment had numerically higher utilization than control participants, including the following: having multiple ED visits, at least 1 ED visit for injury, visits to multiple EDs, being high-frequency utilizers (≥4 ED visits in 12 months), return ED visits within 3 days, return ED visits within 7 days, ED visits for ACSCs, and low-urgency ED visits.

Some of these differences in utilization did not reach statistical significance. Low-urgency ED visits in the 12 months before mistreatment identification were similar between case patients and control participants. Notably, ED utilization for older adults experiencing mistreatment was much higher in the 12 months after identification than before, leading to more pronounced differences between case patients and control participants in utilization postidentification. Emergency department visits for case patients were significantly more likely to have led to hospitalization over the 24-month period surrounding mistreatment identification (82 [36.1%] vs 118 [28.3%]; AOR, 1.99 [95% CI, 1.17-3.37]; *P* = .01), particularly during the 12 months prior to mistreatment identification (35 [42.7%] vs 56 [27.3%]; AOR, 3.32 [95% CI, 1.15-9.57]; *P* =.03).

**Figure 3.**
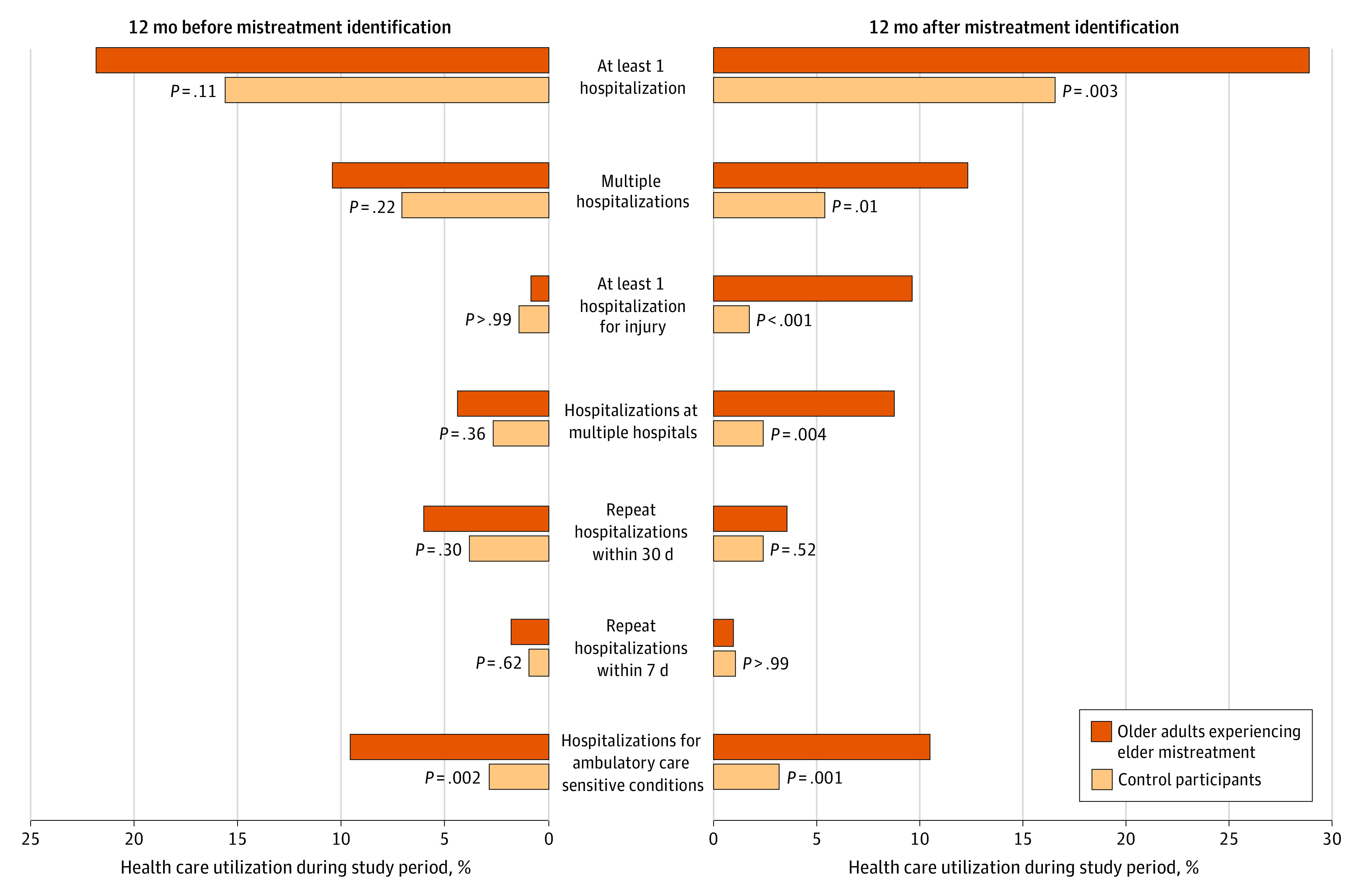
Hospital Utilization Among 114 Older Adults Known to Be Experiencing Elder Mistreatment Compared With 410 Matched Control Participants

Figure 3 depicts the comparison of 7 measures of hospitalization during these same periods. It shows that older adults experiencing mistreatment were significantly more likely to have been hospitalized in all periods compared with controls. Although less likely to be hospitalized for injury in the 12 months prior to mistreatment identification and with a similar frequency of repeat hospitalizations within 7 days in the 12 months including and after mistreatment identification, older adults experiencing mistreatment had higher levels of hospital utilization by all other measures during all periods under study compared with control participants. Similar to ED utilization, hospital utilization was much higher for older adults experiencing mistreatment during the year after mistreatment identification, with larger differences between cases and controls.

### Adjustment for HCC Score

The AORs (after adjusting for CMS-HCC score) comparing the case vs control groups for ED and hospital utilization are reported in the Table. The AORs for most measures of utilization were still significantly higher for elder mistreatment case patients than for matched control participants. During the 24 months surrounding mistreatment identification, older adults experiencing mistreatment were more likely to have had an ED visit (77 [67.5%] vs 179 [43.7%]; AOR, 2.95 [95% CI, 1.78-4.91]; *P* < .001) and more likely to have been hospitalized (44 [38.6%] vs 108 [26.3%]; AOR, 1.90 [95% CI, 1.13-3.21]; *P* = .02) compared with other older adults. Notably, during the 12 months after mistreatment identification, older adults experiencing mistreatment were particularly more likely to have had high-frequency ED use (12 [10.5%] vs 8 [2.0%]; AOR, 8.23 [95% CI, 2.56-26.49]; *P* < .001) and to have visited the ED for low-urgency issues (12 [10.5%] vs 8 [2.0%]; AOR, 7.33 [95% CI, 2.54-21.18]; *P* < .001).

**Table.** Health Care Utilization Among 114 Older Adults Known to Be Experiencing Elder Mistreatment Compared With 410 Matched Controls

Utilization type	720 d Surrounding identification		360 d Prior to identification		360 d Postidentification	
	AOR (95% CI)	*P* value	AOR (95% CI)	*P* value	AOR (95% CI)	*P* value
ED visit						
≥1	2.95 (1.78-4.91)	<.001	1.68 (1.03-2.74)	.04	2.74 (1.74-4.31)	<.001
Multiple	2.59 (1.57-4.27)	<.001	1.97 (0.97-4.00)	.06	2.92 (1.64-5.23)	<.001
≥1 for injury	2.37 (1.43-3.93)	<.001	1.32 (0.63-2.78)	.46	2.96 (1.59-5.52)	<.001
To multiple EDs	2.01 (1.14-3.57)	.02	1.56 (0.54-4.50)	.41	4.14 (1.86-9.22)	<.001
High frequency (≥4 visits annually)	4.45 (2.05-9.67)	<.001	2.66 (0.67-10.65)	.17	8.23 (2.56-26.49)	<.001
Return within 3 d	2.12 (0.72-6.17)	.17	2.66 (0.48-14.77)	.26	1.85 (0.45-7.56)	.39
Return within 7 d	2.79 (1.17-6.66)	.02	2.43 (0.68-8.68)	.17	2.17 (0.81-5.85)	.12
For ACSCs	1.97 (0.90-4.31)	.09	1.22 (0.39-3.82)	.74	1.78 (0.68-4.63)	.24
Low urgency	3.64 (1.73-7.66)	<.001	1.15 (0.31-4.26)	.84	7.33 (2.54-21.18)	<.001
Leading to hospital admission	1.99 (1.17-3.37)	.01	3.32 (1.15-9.57)	.03	1.52 (0.74-3.11)	.26
Hospitalization						
≥1	1.90 (1.13-3.21)	.02	1.63 (0.80-3.34)	.18	2.30 (1.37-3.88)	.002
Multiple	3.07 (1.53-6.16)	.002	1.47 (0.47-4.63)	.51	3.25 (1.45-7.30)	.004
≥1 for injury	6.64 (2.24-19.68)	<.001	0.81 (0.07-8.81)	.86	8.53 (2.46-29.55)	<.001
At multiple hospitals	3.15 (1.34-7.38)	.008	2.85 (0.54-15.12)	.22	4.69 (1.59-13.80)	.005
Repeat within 30 d of discharge	2.36 (0.82-6.85)	.11	2.39 (0.61-9.44)	.21	1.84 (0.50-6.81)	.36
Hospitalization for ACSCs	2.93 (1.34-6.40)	.007	3.15 (1.14-8.69)	.03	3.57 (1.44-8.81)	.006

Abbreviations: ACSC, ambulatory care sensitive condition; AOR, adjusted odds ratio; ED, emergency department.

### Utilization During the 4-Month Subperiods

**Figure 4.**
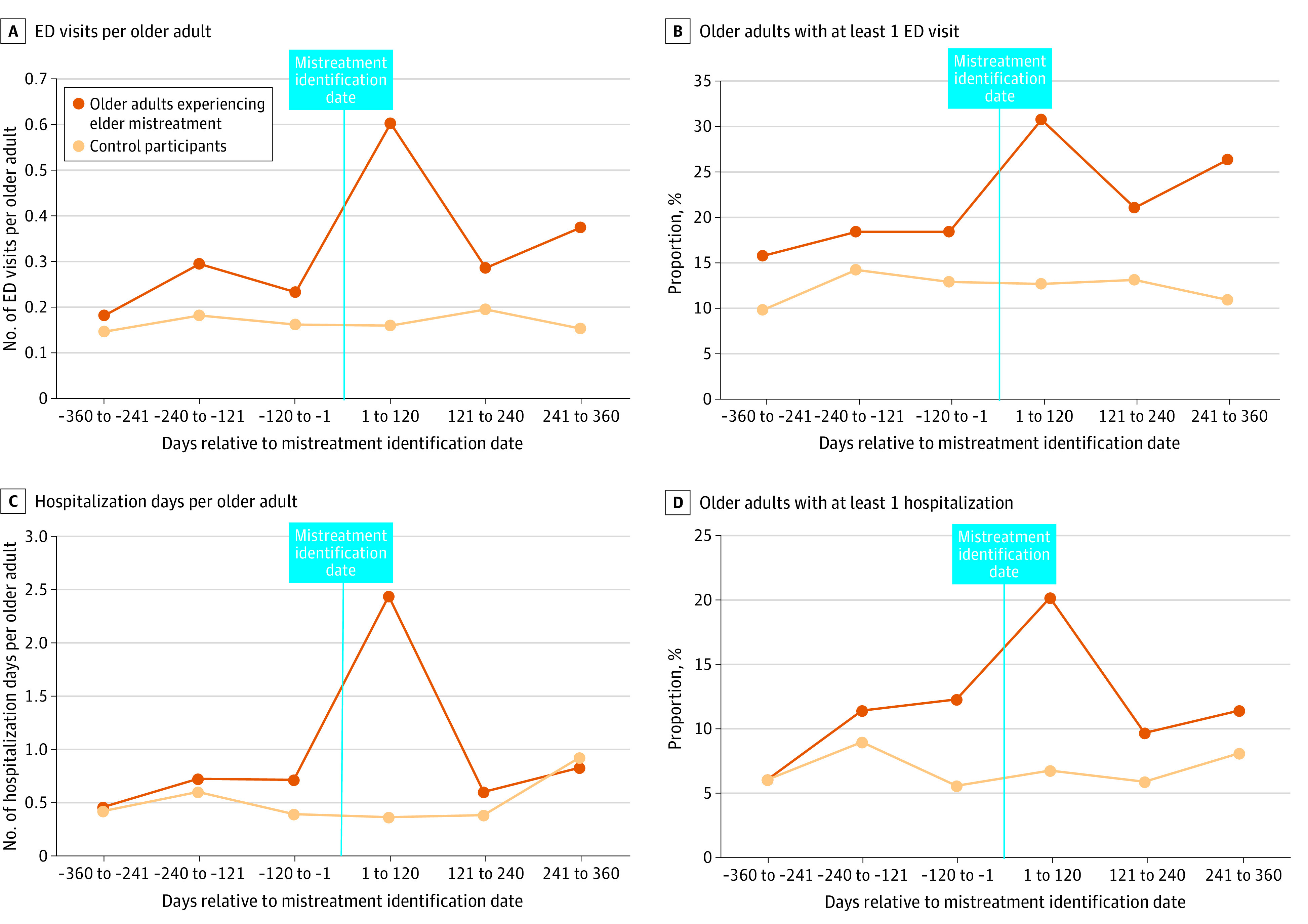
Emergency Department and Hospital Utilization of 114 Older Adults Known to Be Experiencing Elder Mistreatment in the Periods Surrounding Mistreatment Identification Compared With 410 Matched Control Participants A to D, Number of emergency department (ED) visits (A), older adults with at least 1 ED visit (B), hospitalization days (C), and older adults with at least 1 hospitalization (D).

Unadjusted ED and hospital utilization during the 4-month subperiods is depicted in Figure 4, which shows that both ED and hospital utilization were highest during the identification/acute postidentification period (0-120 days after mistreatment identification). Within the 12 months prior to mistreatment identification, the periods with the highest utilization were the acute (−120 days to −1 day from mistreatment identification) and subacute (−240 to −121 days from mistreatment identification) preidentification periods.

## Discussion

This case-control study represents, to our knowledge, the first detailed examination of characteristics of ED and hospital utilization among older adults experiencing elder mistreatment over an extended time period surrounding mistreatment identification. As we hypothesized, the results of this study suggest that older adults experiencing mistreatment used the ED and hospital more frequently compared with other older adults. This finding was observed across multiple measures of utilization. In addition to increased frequency, older adults experiencing mistreatment were more likely to use the ED and hospital in nonoptimal ways, including multiple visits, high frequency of utilization, visits to multiple institutions, repeat ED visits at short intervals, ED visits and hospitalizations for ACSCs, and low-urgency ED visits. Although not all differences were statistically significant, point estimates suggested that they were potentially clinically important. This observation suggests that in addition to acute care needs directly associated with their mistreatment, older adults experiencing mistreatment may be less connected to primary and outpatient care, leading to exacerbation of health conditions that then require acute, unscheduled care.^18^

This pattern of high-intensity, high-cost utilization confirms and expands on previous work suggesting increased utilization among older adults experiencing elder mistreatment.^2,5,17^ Notably, in this study, this increased utilization was much more pronounced after mistreatment identification. It is possible that mistreatment identification led to recognition of other medical issues that required acute management. This offers an opportunity for potential intervention in the ED and/or hospital for older adults known or suspected to be experiencing mistreatment. This intervention may be coordinated by team members from multiple disciplines and may involve ensuring safety, connecting older adults to community-based programs and resources, strengthening existing social supports, offering respite care for an overwhelmed caregiver, identifying other unmet needs, and reporting to the department of adult protective services as appropriate.^40,41,42^ Initiating intervention may be deeply impactful even for older adults experiencing nonphysical mistreatment, including financial exploitation and verbal, emotional, or psychological abuse, given the impact that this mistreatment can have on an older adult’s quality of life. Additionally, the ED or hospital may ensure that older adults have access to outpatient care and that outpatient clinicians are more proactive in treating these patients to minimize acute unscheduled ED or hospital care.

During the 12 months before mistreatment identification, ED and hospital utilization was highest in the acute preidentification period. It is likely that in many cases, mistreatment was already occurring and perhaps escalating during this period, leading to increased health care utilization and the likelihood of identification. These increased ED visits and hospitalizations during the 4 months before identification suggest that some of these older adults experiencing elder mistreatment could have been identified earlier. This finding emphasizes the importance of assessment and screening for elder mistreatment in EDs and hospitals.^43^ In the future, machine learning analysis of health care utilization data may contribute to clinical decision support tools to assist in early identification.^44^

That ED visits for older adults experiencing elder mistreatment were more likely to lead to hospitalization suggests either that older adults experiencing elder mistreatment presented with more severe medical problems or that medical and/or social issues prevented them from being safely discharged from the ED. This observation was most pronounced during the year before mistreatment identification, suggesting further opportunities to identify mistreatment earlier. The results highlight the increased use of high-intensity, high-cost health care services by older adults experiencing mistreatment. These findings suggest that older adults experiencing mistreatment are likely to incur higher health care costs than other older adults, underscoring the financial burden associated with this phenomenon.

### Limitations

This case-control study has several limitations that point toward directions for future research. Because of the need to link cases to Medicare data and for 24 months of continuous enrollment in fee-for-service Medicare surrounding mistreatment identification, we were unable to include most of the elder mistreatment cases from our study cohorts. It is possible that cases not included may have had different health care utilization than those that were studied. Additionally, the cases we examined had elder mistreatment that was identified and successfully adjudicated by the legal system. Such individuals represent a small percentage of all cases, and the older adults analyzed here may have experienced more acute or severe mistreatment allowing for identification. Less intensive cases of mistreatment that are more challenging to detect and prosecute may not have been included. Cases examined in this article also may reflect older adults with a better support system (compared with those with similar experiences of mistreatment but not identified and adjudicated), increased ability to report mistreatment, and better health care and legal literacy.

By examining many outcomes, we may have increased the potential for type I error. Our goal, however, was to comprehensively describe utilization using established measures. The small sample size prevented us from examining differences in utilization patterns between types of mistreatment or in subpopulations of cases. Future research with a larger sample would be helpful to provide insight. Given that in many cases the elder mistreatment triggering identification also led directly to an ED visit and potential hospitalization, some of the additional utilization that we found could be explained by this. We conducted a sensitivity analysis by excluding ED visits and hospitalizations on the day of mistreatment identification, however, and our findings of increased utilization persisted. Furthermore, this research relied on the evaluation of administrative data, which was designed for billing rather than research and may have incomplete or inconsistent information. Despite these limitations, this innovative methodology paves the way for future studies.

## Conclusions

The findings of this study suggest that older adults experiencing elder mistreatment use EDs and hospitals more frequently than other older adults and more commonly in nonoptimal ways during the period surrounding mistreatment identification, particularly after identification. Future research is needed to better characterize these different patterns, which may be helpful in informing early identification, intervention, and prevention.

## Notes

Supplement.
Data Sharing Statement
