# Supplementary material for: Emergency Department and Hospital Utilization Among Older Adults Before and After Identification of Elder Mistreatment
Source: JAMA Netw Open. 2023 Feb 14;6(2):e2255853. doi: 10.1001/jamanetworkopen.2022.55853 (PMC9929702; doi:10.1001/jamanetworkopen.2022.55853)
Supplement: Supplement. — Data Sharing Statement [file jamanetwopen-e2255853-s001.pdf]

Data Sharing Statement

Rosen. Emergency Department and Hospital Utilization Among Older Adults Before and After Identification of Elder Mistreatment. *JAMA Netw Open*. Published February 14, 2023. doi:10.1001/jamanetworkopen.2022.55853

Data
Data available: No

Additional Information
Explanation for why data not available: Data used for this research was medicare claims data access through Virtual Research Data Center. Therefore, this data cannot be made available to others.
